# Green Synthesis of Ag/AgCl Nanoparticles From Argan Oil Waste: Characterization, Antibacterial, and Cytotoxicity Activities

**DOI:** 10.1002/fsn3.71317

**Published:** 2025-12-12

**Authors:** Asma El Kaourat, Hicham El Mouahid, Noura Bentarhlia, Badr Eddine Kartah, Yassine Jaouhari, Asmaa Oubihi, Aicha Guessous, Matteo Bordiga, Hanae El Monfalouti

**Affiliations:** ^1^ Laboratory of Plant Chemistry, Organic and Bioorganic Synthesis, Faculty of Sciences Mohammed V University in Rabat Rabat Morocco; ^2^ Laboratory of Materials, Nanotechnology and Environment, Faculty of Sciences Mohammed V University in Rabat Rabat Morocco; ^3^ Laboratory of Natural Resources and Sustainable Development, Department of Biology, Faculty of Science Ibn Tofail University Kénitra Morocco; ^4^ Department of Pharmaceutical Science Università Degli Studi del Piemonte Orientale “A. Avogadro” Novara Italy

**Keywords:** Ag/AgCl‐NPs, antibacterial, argan press cake, cytotoxicity, saponin extract

## Abstract

Green synthesis of metal nanoparticles (NPs) from biological sources provides a sustainable and biocompatible approach with diverse applications in biomedicine, catalysis, and environmental remediation. In this study, silver/silver chloride nanoparticles (Ag/AgCl‐NPs) were synthesized using saponins extracted from argan press cake. The nanoparticles were characterized using UV–visible spectrophotometry, Fourier‐transform infrared spectroscopy (FTIR), X‐ray diffraction (XRD), scanning electron microscopy coupled with energy‐dispersive X‐ray spectroscopy (SEM/EDS), and transmission electron microscopy (TEM). The initial indication of nanoparticle formation was a yellow coloration, with a distinct absorption band observed between 400 and 450 nm in the UV–visible spectrum. TEM analysis revealed that the nanoparticles were spherical, with an average diameter of 12.3 ± 7.6 nm. The antibacterial and cytotoxic properties of the Ag/AgCl‐NPs were also evaluated. Antibacterial activity was assessed against Gram‐positive and Gram‐negative pathogenic bacteria using disk diffusion, minimum inhibitory concentration (MIC), and minimum bactericidal concentration (MBC) assays. In vitro cytotoxicity was tested against MDA‐MB‐468, MCF‐7, and HeLa cancer cell lines. The results demonstrated that the biosynthesized Ag/AgCl‐NPs exhibited strong antibacterial activity and moderate cytotoxic effects, indicating their potential for biomedical applications.

## Graphical abstract

Ag/AgCl nanoparticles were biosynthesized using argan‐derived saponins and characterized by UV–Vis, FTIR, XRD, SEM/EDS, and TEM, confirming spherical structures averaging 12.3 ± 7.6 nm. The nanomaterials displayed a characteristic surface plasmon resonance at 400–450 nm and crystalline Ag/AgCl phases. Antibacterial performance against Gram‐positive and Gram‐negative strains was verified through disk diffusion, MIC, and MBC assays, while cytotoxicity was demonstrated in MDA‐MB‐468, MCF‐7, and HeLa cell lines. The results support their potential as sustainable nanomaterials for biomedical applications.

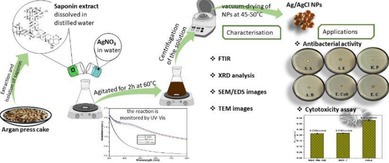

## 1 Introduction

Bio‐nanotechnology is an emerging and innovative discipline that focuses on the production of nanoparticles derived from biological sources. It has achieved notable success in therapeutic and healthcare applications (Chikkanayakanahalli Paramesh et al. 2021; Pirtarighat et al. 2019) and has found relevance across multiple sectors, including medicine, biomedicine, food, cosmetics, textiles, agriculture, parasitology, water treatment (Chaloupka et al. 2010; Le et al. 2020; Politano et al. 2013). In recent years, there has been a significant increase in the utilization of medicinal plants due to their natural healing properties and minimal adverse effects. The synthesis of silver nanoparticles (AgNPs) using plant extracts, bacteria, and fungi represents a green chemistry approach that is environmentally friendly and cost‐effective, contributing to the advancement of sustainable chemical processes (Ndikau et al. 2017).

Despite its advantages, the biosynthesis of nanoparticles presents certain limitations, including multi‐step synthesis procedures, dependence on toxic solvents, external halide sources, and prolonged reaction times (Devi et al. 2016). Typically, noble metals such as silver, platinum, gold, zinc, and palladium are employed in nanoparticle synthesis. However, traditional physical, chemical, photochemical, electrochemical, and radiation‐based synthesis methods may pose risks in medical applications, underscoring the necessity for environmentally sustainable synthesis techniques, which have become a key focus of green chemistry (Azizi et al. 2014).

Silver nanoparticles (AgNPs) have demonstrated high efficacy in combating a range of severe diseases, particularly those caused by multi‐drug‐resistant pathogens (Rahmaniyan et al. 2015). They exhibit antibacterial, antifungal, and anticancer activities, showing non‐cytotoxic effects on human cells while maintaining effectiveness against fungi, viruses, and bacteria at low concentrations (Veerasamy et al. 2011). The growing problem of antimicrobial resistance further underscores the urgent need for novel antimicrobial agents, thereby enhancing the significance of research on medicinal plant (Philip et al. 2011).

Nanoparticles offer superior biocompatibility compared to conventional drugs, enabling more precise and targeted delivery. This enhanced specificity improves therapeutic efficacy while reducing adverse side effects, As a result, it positions AgNPs as promising drug candidates (Y. Li et al. 2006). Metal nanoparticles exhibit diverse biological properties including antibacterial, antiviral, anticancer, thrombolytic and anticoagulant effects, making them invaluable in diagnosis and imaging. Among them, silver nanoparticles stand out for their exceptional versatility; they can be integrated into composite fibers, cryogenic superconductors, and used in electronic devices, cosmetics and food products. Additionally, AgNPs demonstrate notable antiviral, antibacterial, antifungal and anti‐inflammatory properties (Austin et al. 2014; Zulfiqar et al. 2024).

Plant‐derived AgNPs have shown enhanced biological activities, functioning as antioxidants (Moldovan et al. 2016), and exhibiting anticancer, anti‐inflammatory (Hasan et al. 2022), antibacterial (Essghaier et al. 2023), and antidiabetic (Jini et al. 2022) properties. Due to their small size, nanoparticles can readily penetrate cellular barriers and disrupt vital biological processes, leading to potential toxicity and dysfunction. Therefore, assessing the biological impacts of nanoparticles is essential to ensure the safe application and continued development of nanotechnologies (Fu et al. 2014; Jallali et al. 2024). The use of plants in nanoparticle synthesis has gained increasing attention. Secondary metabolites such as alkaloids, saponins, flavonoids, tannins and phenolic compounds play a central role in this process. This green synthesis approach promotes the use of biodegradable, plant‐based materials, enabling environmentally safe disposal and reducing long‐term ecological impacts. The improved biocompatibility of such plant‐mediated AgNPs supports sustainable nanomaterial production, while safeguarding human health and environmental integrity (Zulfiqar et al. 2024).

Saponins are a class of phytochemicals, structurally characterized by a steroid or triterpenoid aglycone (sapogenin) linked to one or more oligosaccharide chains via glycosidic bonds (Basu et al. 2015), They are well recognized as natural surfactants (Saxena et al. 2014) and are distributed widely in higher plants as well as in protein‐rich foods such as legumes and oilseeds (Lacaille‐Dubois and Wagner 1996). Saponins have recently attracted particular interest due to their amphiphilic nature and diverse biological activities, especially in pharmaceutical applications (Cheok et al. 2014). *Argania spinosa*, an edible oilseed tree, endemic to Morocco, contains oily kernels that yield the highly valued argan oil, traditionally used in medicine and cosmetics (El Monfalouti et al. 2010). The extraction of argan oil produces substantial quantities of press cake, which is rich in saponins, including even saponins, arganine A to F and Mi‐saponin A, which have been identified (Charrouf et al. 1992).

Although the pharmacological properties of saponins from argan press are well documented, no scientific studies have reported their use in silver nanoparticle synthesis Therefore, this study aims to synthesize Ag/AgCl nanoparticles using saponin extracts from argan press cake for the first time. The synthesized nanoparticles were characterized using ultraviolet–visible (UV–Vis) spectroscopy, Fourier transform infrared (FTIR) spectroscopy, scanning electron microscopy (SEM), transmission electron microscopy (TEM), and X‐ray powder diffraction (XRD). Furthermore, their in vitro cytotoxicity and antibacterial activities were evaluated.

## 2 Materials and Method

### 2.1 Extraction of Saponins From Argan Press Cake

Argan press cake (APC) was collected in 2022 from a cooperative located in the Taroudant region, southwestern Morocco. Prior to extraction, the powdered APC was defatted with n‐hexane (1:3 w/v) using a Soxhlet apparatus for 6 h to remove residual oil. The saponin extraction was performed using the method of (Kwon et al. 2003) with some modifications. In brief, the defatted sample was subjected to extraction using an 80:20 ethanol/water as solvent under magnetic stirring for 2 h at 50°C. The mixture was filtered, and the filtrate was concentrated under reduced pressure using a rotary evaporator at 45°C to one‐third of its initial volume. Subsequently, it was washed twice with *n*‐hexane in a separatory funnel to eliminate residual lipids. The aqueous phase was then partitioned three times with *n*‐butanol (1:1, v/v). The final extract was concentrated via rotary evaporation, dried, and stored at 4°C for further use in the study.

### 2.2 Synthesis of Ag/AgCl Nanoparticles

Silver/silver chloride nanoparticles (Ag/AgCl‐NPs) were synthesized according to (Chikkanayakanahalli Paramesh et al. 2021). The method consisted of the addition of 20 mL of saponin (5 mg/mL) to 50 mL of a solution of 0.003 M aqueous silver nitrate (> 99.8% AgNO_3_) and stirred at 60°C for 2 h. A gradual color change from yellow to dark brown indicated the formation of Ag/AgCl‐NPs. After completion of the reaction, the solution was centrifuged to collect the nanoparticles. The pellet was washed three times with distilled water and once with ethanol to remove unreacted constituents and excess ions. The purified nanoparticles were dried at 4°C–50°C under vacuum to constant weight and stored in airtight amber vials until use.

### 2.3 Characterization of Ag/AgCl‐NPs

The synthesis progress of Ag/AgCl‐NPs was measured by a UV–visible spectrophotometer between 300 and 800 nm. The formation of Ag/AgCl NPs was controlled by measuring the UV–Vis spectra of the reaction mixture (aqueous solution of silver nitrate with saponins extract). UV–Vis spectra were measured on a PerkinElmer double‐beam spectrophotometer operating at a resolution of 2 nm in the range between 300 and 800 nm. The saponins extract and silver nanoparticles produced were characterized by FTIR (Perkin Elmer, Spectrum 100 (USA)). The FTIR spectra were recorded in the 4000 and 400 cm^−1^ wavenumber range. The shape and size of the silver nanoparticles achieved were confirmed by scanning electron microscopy (Quanta 200) and transmission electron microscopy (Talos F200S). The crystalline shape of the synthesized Ag/AgCl nanoparticles was determined by powder X‐ray diffraction analysis using CuKα1 radiation from the X‐ray diffractometer (EMPYREAN, Reflexion‐transmission spinner—MALVERN PANALYTICAL) operating at 45 kV and 40 mA, the diffraction angle of 2θ was varied from 10° to 80°. The PANalytical X'pert Highscore software was used to analyze the XRD data.

### 2.4 Antibacterial Activity

#### 2.4.1 Disc Diffusion Method

The antibacterial activity of Ag/AgCl‐NPs was performed using the Mueller‐Hinton agar diffusion method, as described by (Oubihi et al. 2020). A microbial suspension with an optical density corresponding to 1 McFarland standard was spread evenly on a Mueller‐Hinton agar plate. The nanoparticles were solubilized in dimethyl sulfoxide (DMSO). Sterilized Whatman paper discs, each 6 mm in diameter, were soaked in the nanoparticle solution. These discs were then placed on the surface of the agar plate. The plates were incubated at 37°C for 24 h. After incubation, the presence of a clear, circular zone of inhibition around the discs, where no microbial growth occurred, indicates the antibacterial activity of the nanoparticles.

#### 2.4.2 Minimum Inhibitory Concentration (MIC) and Minimum Bactericidal Concentration (MBC)

The minimum inhibitory concentration (MIC) and Bactericide (BMC) were determined by the microdilution method in solid medium (Tarfaoui et al. 2022). The sample is emulsified by an agar solution at a rate of 0.2%. This allows obtaining a homogeneous distribution of the sample in the medium. Dilutions are prepared in this agar solution of 100, 40, 20, 10, 5, 3.3 and 2 μL mL^−1^. To test tubes each containing 13.5 mL of Muller Hinton solid medium, 1.5 mL of each dilution is aseptically added to give final concentrations of 10, 4, 2, 1, 0.5, 0.33, and 0.2 μL mL^−1^. The contents of each tube are immediately poured into a sterile Petri dish after shaking for 15 s. Controls, containing the culture medium alone.

### 2.5 Cytotoxicity Assay

#### 2.5.1 Cell Culture

The cytotoxic activity of the biosynthesized Ag/AgCl‐NPs was evaluated on three human cancer cell lines: MCF‐7 (breast adenocarcinoma), MDA‐MB‐468 (triple‐negative breast cancer), and HeLa (cervical cancer), using the MTT assay. All cell lines were obtained from the American Type Culture Collection (ATCC, Manassas, USA). Cells were cultured in RPMI 1640 medium supplemented with 5% heat‐inactivated fetal bovine serum (FBS), 1% penicillin G‐streptomycin and 0.2% L‐glutamine. All cell cultures were maintained at 37°C in a humidified atmosphere with 5% CO_2_.

#### 2.5.2 Cytotoxicity Evaluation Toward Tumor Cells

Cell viability was assessed using the MTT (3‐(4,5‐dimethylthiazol‐2‐yl)‐2,5‐diphenyltetrazolium bromide) assay. Following a phosphate‐buffered saline wash, cells were seeded in 96‐well plates at densities of 10,000 and 20,000 cells per well and allowed to adhere overnight. Subsequently, 100 μL of culture medium containing Ag/Ag‐NPs (0–1000 μg/mL) was added to each well. Negative controls received dimethyl sulfoxide (DMSO) at concentrations ≤ 0.2%. After 48 h of incubation, 20 μL of MTT reagent (5 mg/mL) was added to each well and further incubated for 4 h. The medium (150 μL) was then carefully aspirated and replaced with 150 μL of acidified isopropanol (0.04 N HCl). Absorbance was measured at 540 nm using a Multiskan microplate reader.

Viability (%) was calculated as 100 × (Ai/A0), where A0 and Ai represent the optical densities of control and treated cells, respectively. The half‐maximal inhibitory concentration (IC50) was derived via nonlinear regression analysis (Jain et al. 2018).

## 3 Results and Discussion

### 3.1 UV‐ Visible Spectroscopy Analysis

**FIGURE 1:**
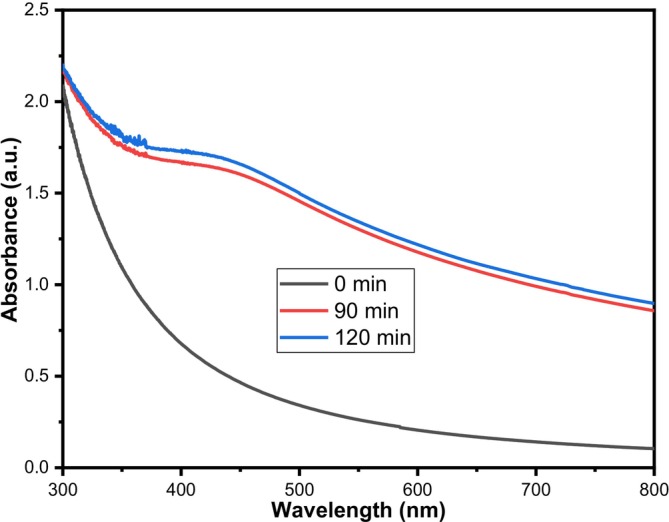
UV–vis absorption spectra of synthesis of silver/silver chloride nanoparticles at different incubated times.

The successful synthesis of Ag/AgCl‐NPs was confirmed by UV–vis spectroscopy. The green synthesis of Ag/AgCl nanoparticles was achieved using saponins extracted from argan press cake. It is well established that Ag‐NPs exhibit characteristic Surface Plasmon Resonance (SPR) absorption in the range 400–500 nm (Bindhu and Umadevi 2014). The UV–Vis spectra presented in Figure 1 show the evolution of the absorption bands at different synthesis times. During the reaction, the color of the mixture gradually changed from white to dark brown, indicating nanoparticle formation. The appearance of distinct absorption peaks between 428 and 430 nm confirmed the formation of Ag/AgCl‐NPs. In contrast, the spectrum of saponins (at 0 min) showed no absorption in this region, further supporting the successful synthesis of the Ag/Ag‐NPs.

### 3.2 X‐Ray Diffraction Analysis

**FIGURE 2:**
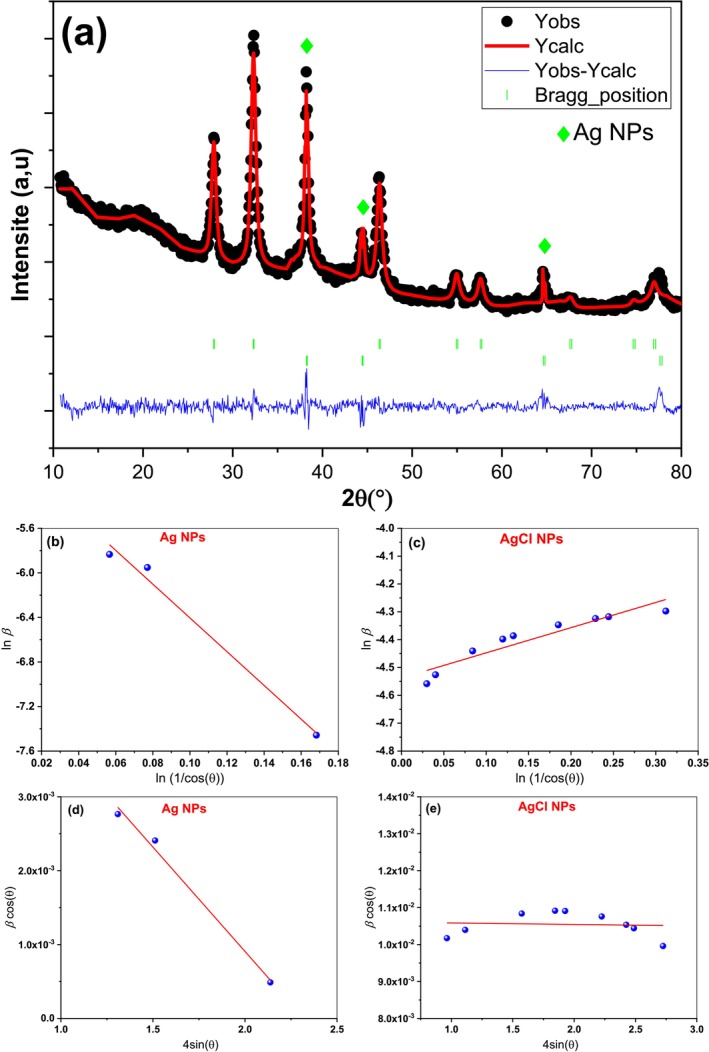
Rietveld refinement of XRD patterns (a), plots of the modified Scherrer model (b, c) and plots of the W‐H method (UDM) (d, e) for silver/silver chloride nanoparticles.

The crystal structure of the dried Ag/AgCl NPs powder was analyzed by XRD. The XRD patterns (Figure 2) revealed five clear and dominant peaks at 38.0°, 44.3°, 64.6°, 77.4° and 81.6° corresponding to the (111), (200), (220), (311) and (222) planes of Ag^0^, respectively. These reflections confirm the formation of a face‐centered cubic (fcc) crystal structure (JCPS No. 04–0783). In addition, distinct peaks observed at 27.9°, 32.3°, 46.3°, 54.8°, 57.5°, 67.7°, 74.5°, 76.9°, and 85.9° were assigned to the (111), (200), (220), (311), (222), (400), (331), (420), and (422) planes of the cubic phase of the AgCl crystal (JCPDS No. 31–1238).

To verify the structural formation of Ag/AgCl NPs, Rietveld refinement was performed using the FullProf software suite. The refinement process involved simultaneous optimization of parameters such as zero correction, scale factor, lattice constants, peak broadening, and thermal factors. The diffraction peak profiles were modeled using a pseudo‐Voigt function, while the background was fitted by linear interpolation between predefined points. The quality of refinement was assessed based on the goodness‐of‐fit indicators and the minimized *χ* ^2^ values (Table 1), with the refined diffraction pattern presented in Figure 2a. The goodness‐of‐fit value plays an important role as an indicator for validating the reliability of the obtained results.

**TABLE 1:** Structural parameters for samples.

Parameters	Ag	AgCl
Crystal System	Cubic	Cubic
Space group	F m^−3^ m	F m^−3^ m
*χ*2	2.123	1.954
Lattice parameter (a) (Å)	4.076262	5.539425
The density (volumic mass) (g/cm3)	5.601	10.578
Weight fraction (%)	45.61	54.39
Lattice volume (Å^3^)	67.731	169.979
Crystallite size (nm) (Debye–Scherrer)	19.26	13.55
Crystallite size (nm) (Williamson‐Hall (W‐H): UDM)	21.94	13.63
Microstrain (Williamson‐Hall (W‐H): UDM)	2.8 × 10^−3^	4.0 × 10^−5^

The results of the Rietveld refinement confirmed that all diffraction peaks of silver and silver chloride were indexed to a cubic structure with the space group F m − 3 m, corresponding to Ag and AgCl phases with weight fractions of approximately 45% and 55%, respectively. Structural parameters, including lattice constants, density, lattice volume are summarized in Table 1. The absence of any additional peaks indicates the high purity of synthesized Ag/AgCl. The presence of sharp and intense diffraction peaks suggests a high degree of crystallinity and the formation of well‐defined nanocrystals of Ag/AgCl (Zhao et al. 2015). This observation is consistent with previous reports on Ag/AgCl nanoparticles synthesized from *C. alata* saponins (Raji et al. 2019). Furthermore, the broader peak with high intensity in the XRD diagram strongly indicates the formation of very small particles with significant crystallinity. Based on the main peaks of the Rietveld refined two‐phase Ag and AgCl data, the crystallite sizes (D) were estimated to be 19.26 and 13.55 nm, respectively, were evaluated from the lnβ_hkl_ vs. ln (1/cosθ) plots that had been plotted (Figure 2b,c) using Scherrer's formula, which is defined as follows (Mandal et al. 2022):

$$\ln\beta_{\mathrm{hkl}}=\ln\left(\frac{\mathrm{K\lambda}}{D}\right)+\ln\left(\frac{1}{\cos\theta }\right)$$

where *K* is a constant equal to 0.94, *λ* is the wavelength of the incident X‐ray (*λ* = 0.154178 nm).

The crystallite size corresponds to the dimensions of a coherently diffracting domain, which may differ from the actual particle size. The broadening of XRD peaks arises from two primary contributions: βhkl = βd + βs, where βd denotes size‐induced broadening (βd = Kλ/Dcosθ) and βs represents strain‐induced broadening (βs ≈ ε/tanθ). In contrast to the Scherrer equation, which exhibits a 1/cosθ dependence, the Williamson–Hall (W–H) method is characterized by its tanθ dependence. For uniform strain distribution across all crystallographic directions, the combined effects of size and strain broadening can be described by the following expression for Bragg reflection line broadening (Kashyap et al. 2024; Mandal et al. 2022; Pormehr et al. 2022):

$$\beta_{\mathrm{hkl}}\cos\theta =\frac{\mathrm{K\lambda}}{D}+4\varepsilon \sin\theta$$

which is known as the uniform deformation model (UDM). Based on the above relationship, linear Williamson‐Hall (W‐H) plots were plotted, as shown in Figure 2d,e. The crystallite sizes of Ag and AgCl are estimated from the y‐intercept values of these plots, giving D_Ag_ = 21.94 and D_AgCl_ = 13.63 nm. The corresponding microstrain (ε) values, obtained from the slops of the lines, were 2.8 × 10^−3^ and 4.0 × 10^−5^ for Ag and Ag/Cl NPs, respectively, as summarized in Table 1. Such lattice strain can serve as a direct or indirect signature of crystal defects and dislocation density within the nanoparticle structure (Barman et al. 2021; Mandal et al. 2022).

### 3.3 FTIR Analysis

**FIGURE 3:**
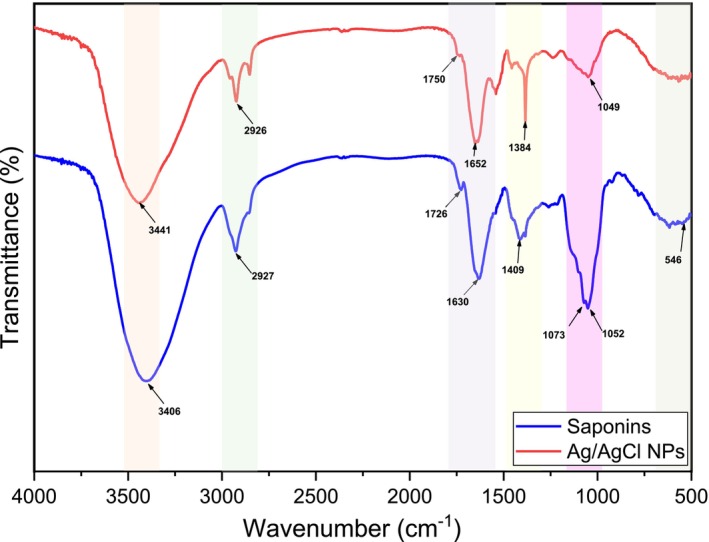
FTIR‐ spectra analyses of argan press cake saponins (blue line) and synthesized Ag/AgCl NPs (red line).

Figure 3 presents the FTIR spectra of saponin extract obtained from argan press cake and of the synthesized Ag/AgCl nanoparticles. The spectrum of saponin (blue line) showed a characteristic infrared absorption band at 3406 cm^−1^ corresponding to the stretching vibration of hydroxyl (OH) groups. The band at 2927 cm^−1^ is attributed to ‐C‐H stretching vibrations, while the bands at 1726 and 1630 cm−1 indicate the C=O and C=C stretching vibrations, respectively. The region from 1446 to 1380 cm^−1^ indicates C‐H deformation vibrations (He et al. 2014). The absorption bands observed between 1034 and 1074 cm^−1^ correspond to oligosaccharide linkage vibrations (C‐O‐C) in sapogenins, and the band at 546 cm^−1^ is due to = C‐Cl bending vibrations in the saponin (Debnath and Das 2019). These spectral features are consistent with previously reported IR spectra of saponins (Almutairi and Ali 2015; He et al. 2014). For the spectrum of Ag/AgCl NPs (red line), the transmittance peaks exhibited changes in intensity and shifted from 3406, 2927, 1726 and 1630 cm^−1^ to 3441, 2926, 1750 and 1652 cm^−1^, respectively. These shifts confirm the formation of coordination bonds between the saponins and Ag on the surface of the nanocrystals (Debnath and Das 2019; Sharifi‐Rad and Pohl 2020). The peak observed at 1384 cm^−1^ corresponds to the NO_3_ vibration (Mandal et al. 2022). This study confirms that the component saponin is responsible for stabilizing Ag/AgCl nanoparticles (Debnath et al. 2020).

### 3.4 SEM and EDS Analysis

**FIGURE 4:**
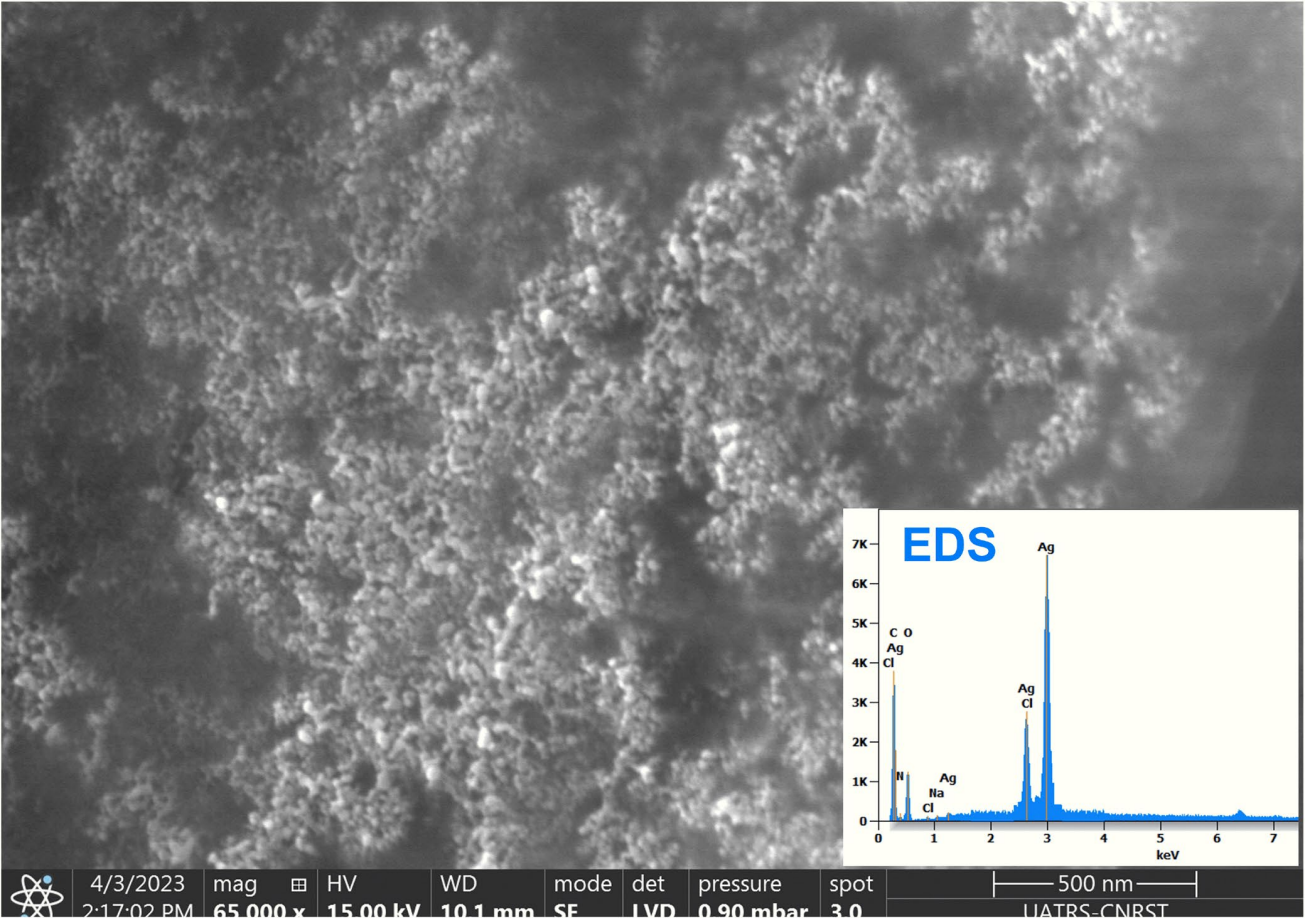
SEM image with magnification ×65,000 and EDS spectra of nanoparticles of Ag/AgCl green synthesized by isolated saponins from Argan press cake.

Figure 4 presents the scanning electron microscopy (SEM) image of the synthesized Ag/AgCl nanoparticles, revealing a predominantly spherical morphology. The energy‐dispersive X‐ray spectroscopy (EDS) analysis confirms the elemental composition, showing a characteristic strong silver signal at 3 keV, along with distinct chloride peaks. These findings verify the successful formation of Ag/AgCl nanoparticles through saponin‐mediated synthesis.

### 3.5 TEM Analysis

**FIGURE 5:**
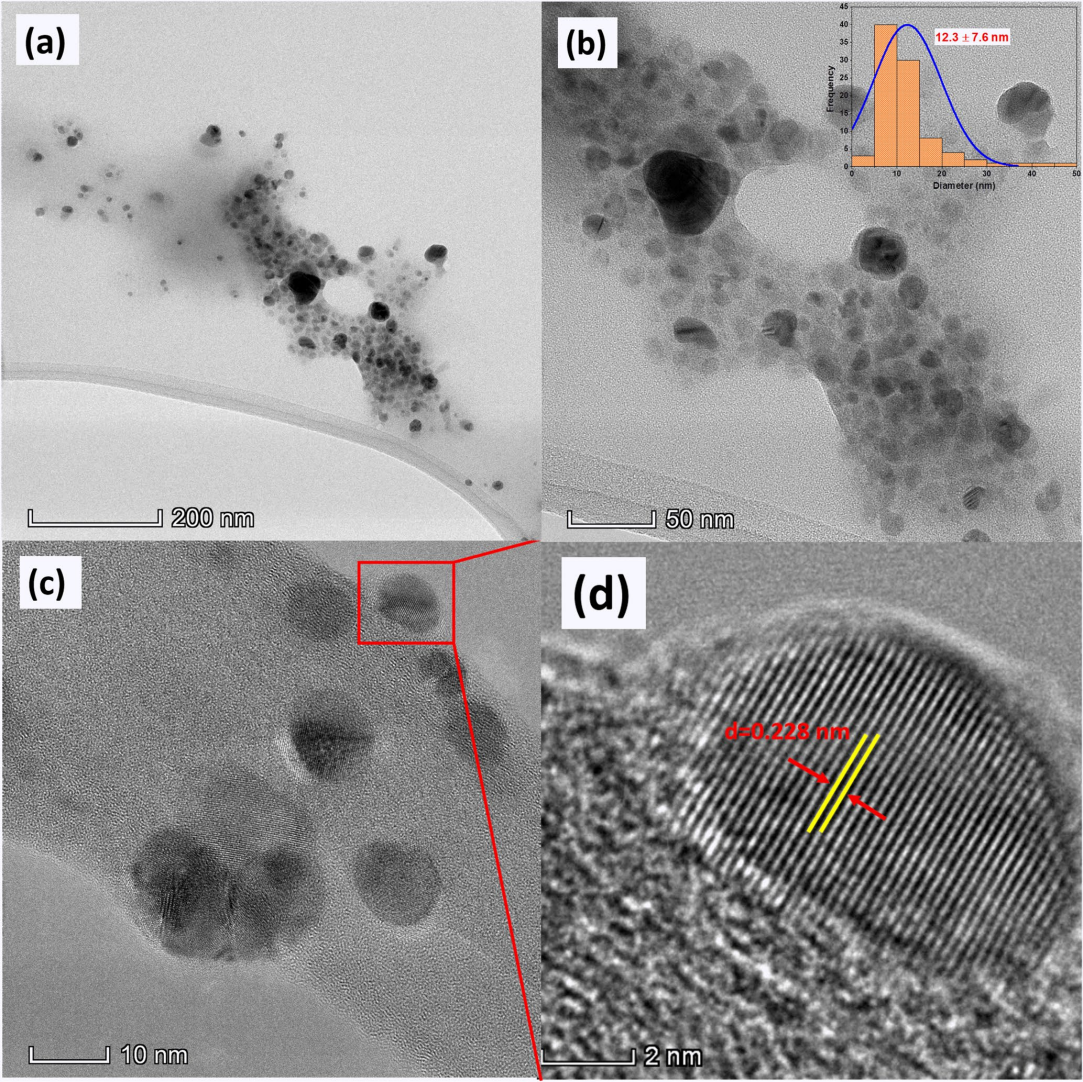
(a, b and c) Transmission electron microscopy (TEM) images of silver nanoparticles (Ag/Ag‐NPs) with the histogram of particle diameter distribution and (d) high‐resolution TEM image of an individual silver nanoparticle.

Typical TEM images of the synthesized Ag/Ag‐NPs are shown in Figure 5a–c at different magnifications. The images show that the synthesized silver nanoparticles are predominantly spherical, with an average size of 12.3 ± 7.6 nm. The corresponding energy‐dispersive X‐ray spectroscopy (EDS) spectrum shows Ag absorption peaks at around 3 keV, confirming the composition of the silver nanoparticles formed (Figure 5) (Xu et al. 2023). Furthermore, high‐resolution TEM (HRTEM) analysis (Figure 5d) showed an interplanar spacing of 0.228 nm, calculated using ImageJ software, which corresponds to the (111) planes of the face‐centered cubic (FCC) structure of silver (Ghosh et al. 2023).

### 3.6 Antibacterial Activity of Ag/AgCl‐NPs

**FIGURE 6:**
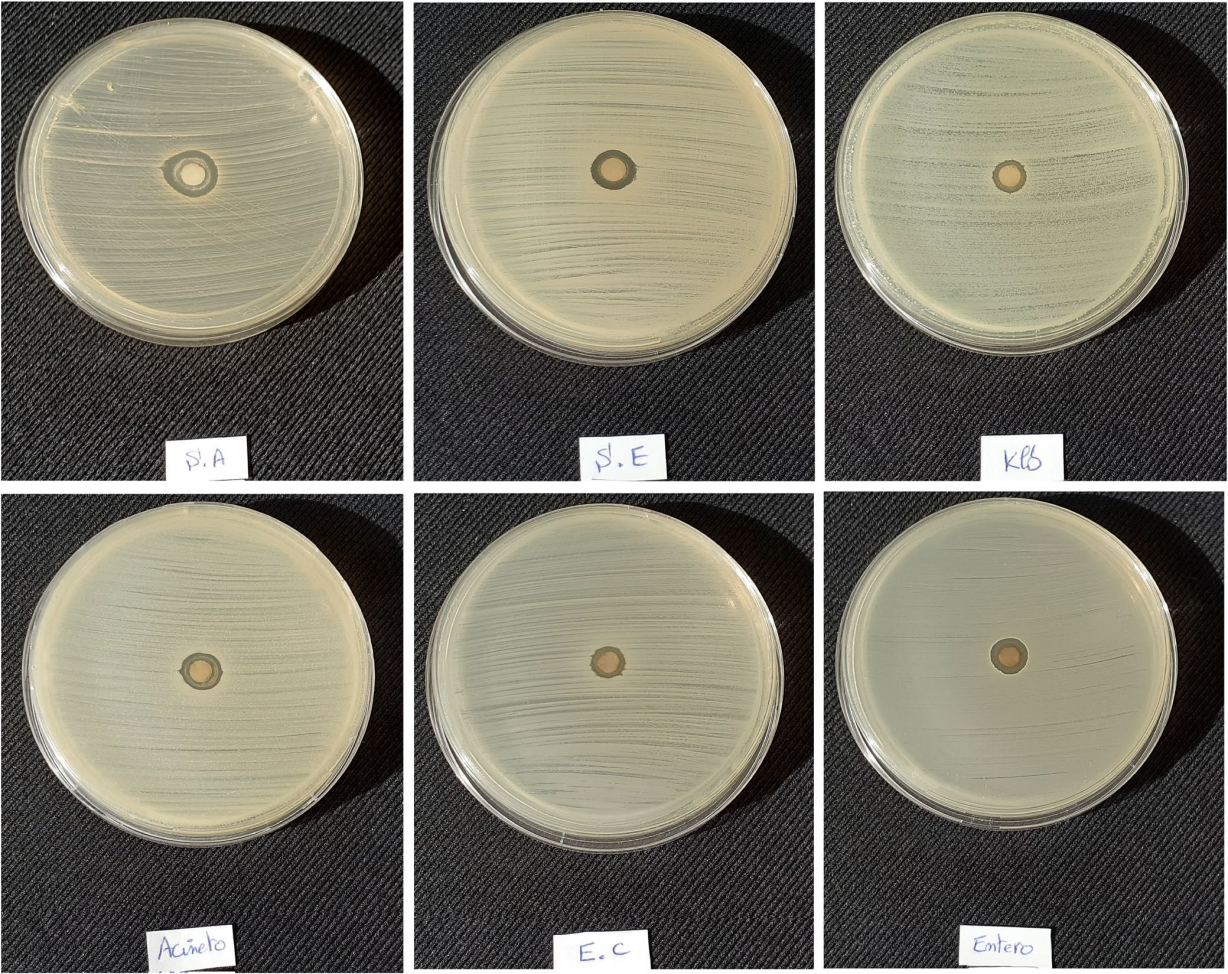
The antibacterial impact of Ag/AgCl‐NPs on both Gram‐negative bacteria and Gram‐positive bacteria.

**FIGURE 7:**
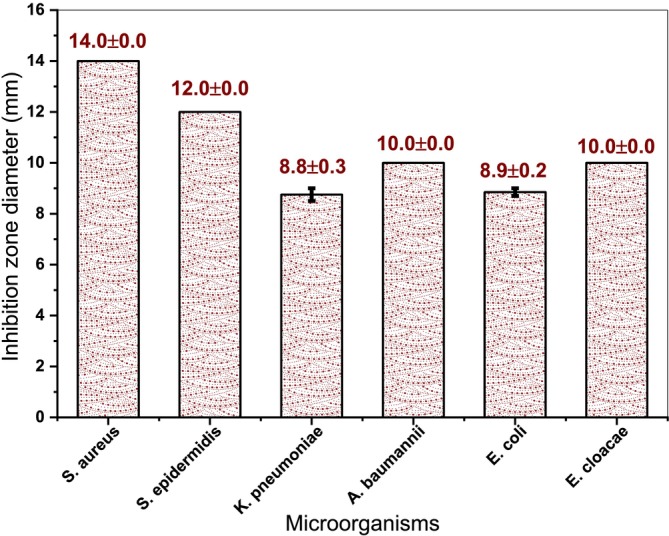
Diameter of the inhibition zone including the disc diameter of 6 mm, by the agar disc diffusion of Ag/AgCl‐NPs/disc.

Antibiotic resistance represents one of the major challenges in modern medical science, primarily arising from the improper or excessive use of antibiotics. With the advancement of nanotechnology, the development of silver nanoparticles (AgNPs) and the demonstration of their strong antimicrobial properties, offer a promising approach to mitigating this problem (Balaraman et al. 2020; Feizi et al. 2018; Satapathy et al. 2017). In this study, the antimicrobial activity of Ag/AgCl nanoparticles synthesized using saponins extracted from argan press cake was evaluated. The antibacterial efficacy was tested against six clinically and foodborne‐relevant bacterial pathogens: two Gram‐positive strains (*Staphylococcus aureus and Staphylococcus epidermidis*) and four Gram‐negative strains (*Klebsiella pneumoniae, Escherichia coli, Acinetobacter baumannii, and Enterobacter cloacae*) (Figure 6). Antimicrobial activity was assessed by measuring the diameters of the inhibition zones. These bacterial species were selected due to their pathogenic relevance and frequent involvement in food contamination (Hajib et al. 2020). As shown in (Figure 7), Ag/AgCl‐NPs showed significant antimicrobial effects against all tested bacterial strains. The inhibition zones indicated that *S. aureus* and *S. epidermidis* were more susceptible to Ag/AgCl‐NPs than the Gram‐negative strains ( *K. pneumoniae* , *E. coli* , *A. baumannii* , and *E. cloacae* ). Interestingly, previous studies have often reported stronger antimicrobial effects of nanoparticles against Gram‐negative bacteria, regardless of their resistance levels, compared to Gram‐positive bacteria (Abo‐Shama et al. 2020; Hassan et al. 2021). The difference observed here may be attributed to variations in cell wall structure; Gram‐negative bacteria possess a complex cell wall structure, containing an outer membrane composed of lipopolysaccharides, which serves as an additional barrier limiting the penetration of antimicrobial agents. In contrast, Gram‐positive bacteria have a simpler cell wall structure dominated by a thick peptidoglycan layer (Rai et al. 2012). The higher antimicrobial efficiency of the Ag/AgCl‐NPs synthesized in this study, compared to previously reported nanoparticles may be attributed to their smaller average particle size of 12.3 ± 7.6 nm, whereas earlier studies reported sizes ranging from 23 to 50 nm (Dong et al. 2014), (Gopinath et al. 2013). Smaller nanoparticles possess a larger surface area‐to‐volume ratio, enhancing their interaction with bacterial cell walls, facilitating penetration, and promoting membrane disruption. This improved contact likely contributes to the enhanced antibacterial efficacy observed. Additionally, the concentration of AgNO_3_ used during synthesis plays a crucial role in determining antibacterial potency, as previously reported by (Salayová et al. 2021).

Ag/AgCl‐NPs showed the best antibacterial activity, as extensively reported (S. Li et al. 2022; Okaiyeto et al. 2019), The MIC was 4 μg/mL, while the MBC was found to be 10 μg/mL for all bacterial strains (Table 2). These results are consistent with those reported by (Hachem et al. 2024), who studied the antibacterial effect of Ag/AgCl‐NPs using *Origanum ehrenbergii Boiss*. They found an MIC value of 62.5 μg/mL and an MBC of around 1000 μg/mL for the same bacterial strains, although other studies have reported different MIC and MBC values for each type of bacterium (Patil et al. 2018; Rolim et al. 2020). This increased efficacy can be attributed to several mechanisms of action. First, the release of Ag^+^ ions into the medium plays a central role, as these ions can bind to bacterial proteins, particularly thiol‐containing enzymes, thereby disrupting essential metabolic processes required for cell viability (Ashraf et al. 2021). Second, the small nanoparticle size (12.3 ± 7.6 nm), facilitates efficient interaction with the bacterial membrane, increasing its permeability and inducing structural destabilization, that leads to loss of integrity and cell death (Pal et al. 2007). Furthermore, the generation of reactive oxygen species (ROS) by Ag/AgCl‐NPs has likely contributed substantially to their bactericidal activity. ROS can cause irreversible oxidative damage to cellular lipids, proteins, and DNA, impairing replication and vital cellular functions (Morones et al. 2005).

**TABLE 2:** MIC and MBC results of Ag/AgCl‐NPs against the representative bacteria.

Microorganisms	MIC (μg/mL)	MBC (μg/mL)
*S. aureus*	4	10
*S. epidermidis*	4	10
*K. pneumoniae*	4	10
*A. baumannii*	4	10
*E. coli*	4	10
*E. cloacae*	4	10

Abbreviations: MBC, Minimum Bactericidal Concentration; MIC, Minimum Inhibitory Concentration.

Although Gram‐negative bacteria, typically exhibit greater resistance due to their outer lipopolysaccharide layer, the small size of the synthesized nanoparticles appears to enable effective penetration of this barrier (Kim et al. 2007; Xiu et al. 2012). In conclusion, the enhanced antibacterial efficacy of the biosynthesized Ag/AgCl‐NPs can be attributed to the synergistic effects of Ag^+^ ion release, membrane disruption, and ROS generation, together with their reduced particle size. These combined mechanisms explain the potent antimicrobial performance observed, particularly against Gram‐positive bacterial strains.

### 3.7 Cytotoxicity Test

**FIGURE 8:**
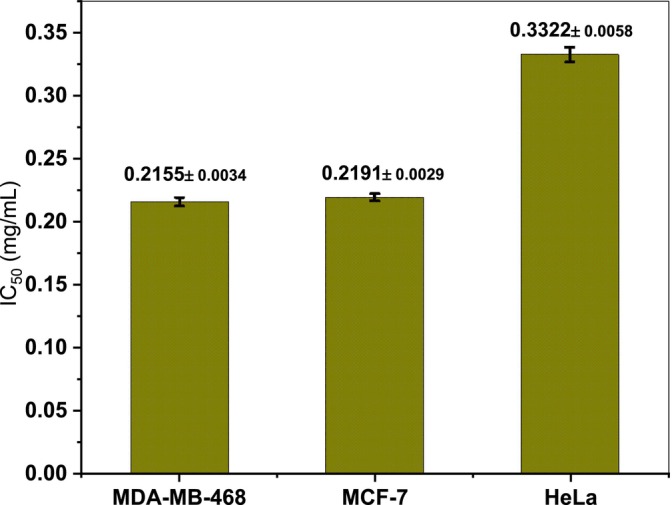
In vitro cell viability of (APC) saponins mediated Ag‐AgCl NPs against MDA‐MB‐468, MCF‐7, HeLa cell line. All data were expressed as mean ± standard deviation (*n* = 3).

Cancer is a complex and severe genetic disease characterized by uncontrolled and abnormal cell division (El‐Naggar et al. 2018). Although chemotherapy and radiotherapy remain the primary approaches for cancer treatment, these methods are often non‐selective, affecting both healthy and malignant cells, and are associated with severe side effects due to the cytotoxic nature of chemotherapeutic agents (Brown 2002). In recent years, the green synthesis of nanoparticles has attracted significant attention in cancer research, particularly for their potential applications in diagnosis and therapy, as well as for evaluating their cytotoxic effects against various cancer cell lines in vitro (Ramkumar et al. 2017). As shown in Figure 8, the biosynthesized Ag/AgCl nanoparticles exhibited potent cytotoxic activity against multiple cancer cell lines, with IC_50_ values of 0.215, 0.219, and 0.332 mg/mL for MDA‐MB‐468, MCF‐7, and HeLa. This cytotoxicity is closely associated with nanoparticle characteristics, particularly their small size and spherical morphology (Ratan et al. 2020). The mechanism underlying this effect is believed to involve the generation of reactive oxygen species (ROS), which induce cellular damage through DNA strand breaks and lipid peroxidation, ultimately impairing essential cellular functions and triggering cell death. Interestingly, metallic nanoparticles can also exhibit antioxidant properties under certain conditions, suggesting a potential dual role in modulating oxidative stress (Hemmati et al. 2020).

The results of the present study confirm that Ag/AgCl nanoparticles possess significant cytotoxic activity against MDA‐MB‐468, MCF‐7, and HeLa. This observed mechanism of action is primarily attributed to the release of Ag^+^ ions into the cellular microenvironment, which interact with the thiol groups of intracellular proteins, disrupting essential enzymatic processes such as DNA replication and protein synthesis, thereby inducing apoptosis (Sun et al. 2021). Additionally, the generation of ROS by Ag/AgCl‐NPs contributes to irreversible oxidative damage to cellular lipids, proteins, and nucleic acids, resulting in profound structural and functional impairments (S. Kim et al. 2009). Cancer cells, owing to their elevated metabolic activity and higher intrinsic oxidative stress, are particularly susceptible to ROS‐mediated damage. Moreover, the small size of the nanoparticles enhances their interaction with cellular membranes and facilitates their internalization, amplifying the cytotoxic effect. In summary, the potent anticancer activity of Ag/AgCl nanoparticles observed in this study can be attributed to the synergistic mechanisms of Ag^+^ ion release, ROS generation, and membrane disruption. These combined effects lead to selective cytotoxicity toward cancer cells while minimizing adverse effects on healthy cells.

## 4 Conclusion

In summary, this study explored the use of saponins extracted from argan press cake (APC) for the green synthesis of silver nanoparticles. The synthesized silver and silver chloride nanoparticles exhibited a face‐centered cubic (FCC) crystal structure with a space group of *Fm–3 m*, and weight fractions of approximately 45% for Ag and 55% AgCl, respectively. The obtained nanoparticles were predominantly spherical, with an average diameter of 12.3 ± 7.6 nm, and demonstrated remarkable antimicrobial activity and selective cytotoxicity toward tumor cell lines. This green synthetic approach not only provides an environmentally friendly, simple, and cost‐effective route for nanoparticle production but also offers a sustainable method for recycling by‐products of the argan oil extraction process. Overall, the findings highlight that saponin‐mediated Ag/AgCl nanoparticles possess significant potential for biomedical applications. Their potent antibacterial and anticancer properties suggest that these nanoparticles could play a vital role in the development of next‐generation therapeutic agents, particularly for combating drug‐resistant pathogens and in cancer treatment. This work therefore provides a promising foundation for future advances in nanobiotechnology and sustainable nanomedicine.

## Author Contributions

**Asma El Kaourat:** writing – original draft, investigation, formal analysis, data curation; **Hicham El Mouahid:** writing – original draft, investigation, formal analysis, data curation; **Noura Bentarhlia:** writing, investigation, data curation; **Badr Eddine Kartah:** review and editing, Resources, Investigation; **Yassine Jaouhari:** data curation, review and editing, validation; **Asmaa Oubihi:** writing, investigation, data curation; **Aicha Guessous:** review and editing, Investigation; **Matteo Bordiga:** review and editing, validation, funding; **Hanae El Monfalouti:** writing – original draft, supervision, formal analysis, conceptualization, validation.

## Funding

This research did not receive any specific grant from funding agencies in the public, commercial, or not‐for‐profit sectors.

## Conflicts of Interest

The authors declare no conflicts of interest.

## Data Availability

The data that support the findings of this study are available on request from the corresponding author. The data are not publicly available due to privacy or ethical restrictions.
